# Effects of far infrared light on Alzheimer’s disease-transgenic mice

**DOI:** 10.1371/journal.pone.0253320

**Published:** 2021-06-17

**Authors:** Koji Fukui, Shunsuke Kimura, Yugo Kato, Masahiro Kohno

**Affiliations:** 1 Department of Bioscience and Engineering, Molecular Cell Biology Laboratory, College of Systems Engineering and Sciences, Shibaura Institute of Technology (SIT), Minato, Japan; 2 Department of Functional Control Systems, Molecular Cell Biology Laboratory, Graduate School of Engineering and Science, SIT, Minato, Japan; 3 Department of Life Science and Technology, Tokyo Institute of Technology, Meguro, Japan; 4 SIT Research Laboratories, The Brain Science & Life Technology Research Center, SIT, Minato, Japan; Nathan S Kline Institute, UNITED STATES

## Abstract

Far infrared light has been used in many medical procedures. However, the detailed biological mechanisms of infrared light’s effects have not yet been elucidated. Many researchers have pointed out the thermal effects of treatments such as infrared saunas, which are known to increase blood flow. Alzheimer’s disease (AD) is associated with gradual decreases in brain blood flow and resulting dementia. In this study, we attempted to clarify the beneficial effects of far infrared light using the 5xFAD mouse, a transgenic model of AD. We exposed 5xFAD mice to far infrared light for 5 months. Among the far infrared-exposed AD mice, body weights were significantly decreased, and the levels of nerve growth factor and brain-derived neurotrophic factor protein were significantly increased in selected brain areas (compared to those in non-irradiated AD mice). However, cognition and motor function (as assessed by Morris water maze and Rota Rod tests, respectively) did not differ significantly between the irradiated and non-irradiated AD mouse groups. These results indicated that exposure to far infrared light may have beneficial biological effects in AD mice. However, the experimental schedule and methods may need to be modified to obtain clearer results.

## Introduction

The electromagnetic wave, which was discovered by Heinrich Rudolf Hertz in 1887, is a wave function that proceeds in a specific direction while crossing electric and magnetic fields. Electromagnetic waves have many characteristics such as reflection, transparency, absorption, refraction, and scattering. Examples of electromagnetic wave applications include the clinical use of X-ray imaging to visualize internal organs, and the heating of objects by the reflection of infrared (IR) waves [1]. Additionally, mobile phone and television systems employ electromagnetic wave-based technologies. Indeed, the use of electromagnetic waves is essential to the comforts of our modern daily life.

Electromagnetic waves are classified by differences in wavelength, which has important effects on their various characteristics. Based on wavelength, waves are characterized as radio waves, microwaves, IR, visible light, ultraviolet, and X-rays [1]. Far IR (FIR) is a type of IR illumination corresponding to wavelengths ranging from 3 to 1000 μm. FIR is known to warm the body with almost no adverse effects, in contrast to ultraviolet rays. As a result, FIR is widely used in heaters, blankets, bedrock baths, and the like. One possible mechanism by which FIR light works is that the absorbed IR rays stimulate molecules arranged in bonded lattices [2], resulting in more violent vibration of the molecules and warming of the substances. It has been proposed that FIR light may have the effect of dilating microvessels, thereby activating blood circulation and strengthening metabolism. However, the mechanism whereby FIR light exerts effects such as heat retention remains unclear.

Alzheimer’s disease (AD) is a severe neurodegenerative disorder, and the number of AD patients is gradually increasing worldwide. The best-known symptom of AD is the attenuation of memory function [3]. The major pathological change associated with AD is the appearance of amyloid plaques and tau hyperphosphorylation in brain tissue [4]. The onset and progression of AD may be related to decreases in brain blood flow [5,6]. The mean age of AD patients typically exceeds 60 years, and the aging process is closely related to AD onset [7]. Because blood capillary dysfunction induces limitations of oxygen supply in the brain and leads to the accumulation of cholesterol and blood clots over time, AD patients often simultaneously exhibit increased risks of arteriosclerosis and cerebrovascular disease [8]. Disorders such as AD, dementia, cardiovascular disease, arteriosclerosis, hypercholesterolemia, hypertension, and diabetes are intricately intertwined. Many factors, such as aging, oxidative damage, and genetic factors, are associated with this complex problem [9]. AD-derived cognitive decline is closely related to alterations of neurotrophic factor levels, such as nerve growth factor (NGF) and brain-derived neurotrophic factor (BDNF) [10], and changes in blood flow [11]. NGF and BDNF play important roles in the maintenance of cognitive functions. Although some reports have indicated that FIR therapy promotes blood circulation [12], the detailed relationship between FIR exposure and AD-related cognitive dysfunction has not yet been elucidated. Here we evaluated the effects of FIR light in a transgenic mouse model of AD. FIR irradiation was provided using a fabric that emits FIR light, and cognitive function and neurotrophic factor protein expressions of FIR-exposed AD-transgenic mice were measured using the Morris water maze and western blotting, respectively.

## Materials and methods

### Animals and reagents

**Fig 1:**
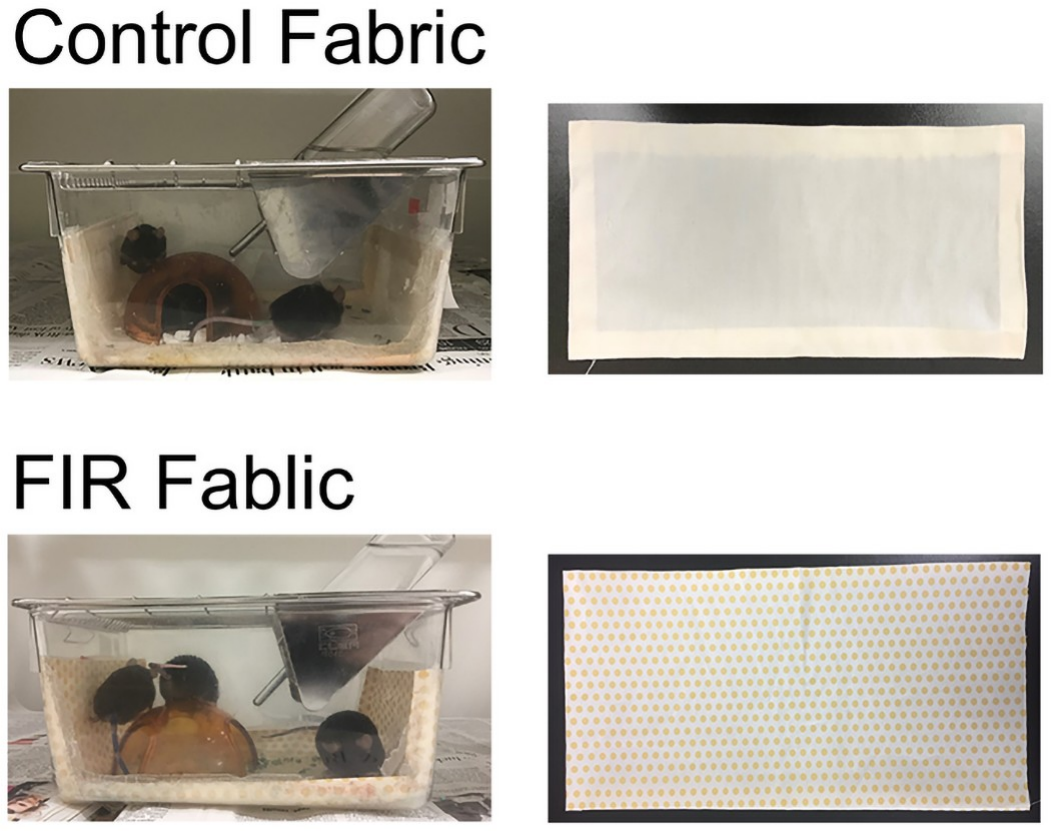
The animal cages with a fabric that generates FIR light or with a control fabric. Sections of fabric derived from the same source cloth were present in both cages, but the fabric in one case was impregnated with a compound that generates far IR light.

All animal experiments were performed with the approval of the Animal Protection and Ethics Committee of the Shibaura Institute of Technology, Tokyo, Japan (Approval number 17006). 5xFAD transgenic mice (#008730, MMRRC034848, B6.Cg-Tg(APPSwFlLon,PSEN1*M146L*L286V)6799Vas/Mmjax, Alias/5XFAD) were purchased from The Jackson Laboratory (Bar Harbor, ME, USA) and self-bred prior to use in these experiments. Eight-month-old AD-transgenic mice were used in this study. Following entry into the study, these mice were maintained for a 5-month period in the presence of a section of fabric that generates FIR light (AD-FIR, n = 6) or in the presence of a section of a similar fabric that does not emit in the FIR range (AD, n = 6) (Fig 1). C57BL/6 male mice of the same age were purchased from Sankyo Labo Service Corp. Inc. (Tokyo, Japan) and used as control groups in the presence or absence of FIR fabric. (Control, n = 5, Control-FIR, n = 5) All mice were maintained under conditions of controlled temperature (22 ± 2°C), a 12-h light/dark cycle, and were provided with free access to food and water. Food consisted of normal diet pellets (Labo MR Stock) purchased from Nosan Corp. (Kanagawa, Japan). The body weights and weights of consumed food (food intake) were measured once per week, and the relative body weights (normalized to the starting value for the respective animal) were calculated from the data. After the treatment period, cognition and motor function were assessed using tests as described below. Following assessments, blood (for serum) was collected from each mouse, mice were euthanized, and samples of brain (cerebral cortex (Cortex), cerebellum (Cer), and hippocampus (Hip)) were collected for analysis. All other chemical agents were obtained from FUJIFILM Wako Pure Chemical Corp. (Osaka, Japan).

### Exposure of AD mice to FIR light

Fabric with ores that emit FIR light was a gift of PMC Co., Ltd. (Ishikawa, Japan; product name: Alpha Slim; U.S. FDA registration number: 30 in 09747901). The fabric swatches were replaced once per week throughout the 5 months of the study. To ensure proximity between the fabric and the animals, the volume of crumpled paper, which is typically used as a flooring material, was decreased from the usual amount, and AD control animal cages were supplied with the same fabric lacking the ore additive. In pilot studies, we tested multiple variations of how the fabric was placed in the animal cages, in order to prevent gnawing on the fabric by the animals. Each cage also was provided with an enrichment tool. The radiation emission of the ores was measured by Toyama Industrial Technology Center using a FIR spectroradiometer (#JIR-E500, JEOL Ltd.) (Measurement No. 832, 1/17/2014). The highest emission wavelength was between 8 to 10 μm, and it produced about 1 W/cm^2^/sr.

### Cognitive performance

Cognitive functions were assessed by Morris water maze and Rota Rod tests, as described previously [13,14] with some modifications. Tests were administered using equipment purchased from Muromachi Kikai Co., Ltd. (Tokyo, Japan).

The Morris water maze apparatus (140 cm in diameter and 45 cm in height) consisted of a pool constructed of acrylic resin. The bottom of the pool was divided into four quadrants by lines and was set up with four different visible marks positioned around the pool. A submerged platform was placed in the center of one quadrant. The water temperature of the pool was maintained at 22 ± 2 °C. Before starting the cognitive performance trials, the animals were acclimated to the pool and to handling by the experimenter by being allowed to swim freely for 60 s in the absence of a platform and by being handled over a 3-day period. The cognitive trials were performed 4 times per day over an interval of 5 consecutive days. All trials were performed at the same time of day and were carried out every 3 h (starting at 10:00, 13:00, 16:00, and 19:00). Thus, we performed a total of 20 trials per mouse. The platform was maintained in the same location of the pool for all trials. The escape latency (time to reach the goal), swimming distance, swimming speed, and the proportion of time spent swimming in the quadrant containing the platform were measured using ANY-maze software (version 6.19; Stoelting Co., Wood Dale, IL, USA).

After finishing the water maze test, we performed the Rota Rod test the next day. The Rota Rod speed was set to accelerate from 5 to 50 rpm over 120 s and the time and speed (in rpm) required for the animal to fall from the Rota Rod were measured. Before starting the Rota Rod study, all mice were allowed to walk for 1 min at the same rod speed (5 rpm). After 1 min, the Rota Rod speed gradually accelerated up to 50 rpm for 2 min, and the rod speed was maintained until the mice fell off the bar. The trials were performed three times and were conducted every 20 min. All tests started at 10:00 and were performed in order.

### Blood analysis

Prior to euthanasia, blood was collected without an anti-coagulant. Following clotting (approximately 30 min at room temperature (RT)), the samples were centrifuged in a clinical centrifuge, and the resulting serum supernatants were transferred to fresh tubes, flash frozen, and stored at -80°C until analysis. The samples were assessed for selected clinical chemistry parameters (including total protein, albumin, aspartate aminotransferase (AST), alanine aminotransferase (ALT), alkaline phosphatase (ALP), lactic acid dehydrogenase (LDH), and others) at an external testing agency (Oriental Yeast Co., Ltd., Tokyo, Japan).

### Western blotting

All brain samples were homogenized in phosphate-buffered saline (PBS) and used for western blotting as described previously [15], with some modifications. Following homogenization, sample lysates were centrifuged; the resulting supernatants (protein extracts) were used for the blotting, and protein contents were determined using the Bradford assay (Bio-Rad protein assay, #500-0006JA, Bio-Rad Laboratories, Inc., Hercules, CA, USA) according to the manufacturer’s protocol. Aliquots of the protein extracts corresponding to 10 μg total protein each were separated on 10% sodium dodecyl sulfate (SDS) -polyacrylamide gels and transferred to nitrocellulose (NC) membranes (ClearTrans; FUJIFILM Wako Pure Chemical Corp.). First, the NC membranes were stained with Ponceau S solution (Merck KGaA, Darmstadt, Germany) and imaged. Next, the NC membranes were washed and incubated in blocking solution (Tris-HCl-buffered saline, pH 7.6 (TBS), containing 0.1% Tween 20 and 2% non-fat skim milk) for 1 h at RT. Following blocking, the membranes were washed at RT in TBS containing 0.1% Tween 20, and then incubated overnight at 4 °C with each primary antibody. The primary antibodies were as follows: rabbit recombinant monoclonal anti-brain-derived neurotrophic factor (BDNF) antibody [EPR1292], 1:2500 (#ab108139, Abcam plc., Cambridge, UK); rabbit polyclonal anti-nerve growth factor (NGF) antibody (H-20), 1:500 (#sc-548, Santa Cruz Biotechnology Inc. (SCBT), Dallas, TX, USA); rabbit polyclonal anti-tropomyosin receptor kinase (Trk) A antibody (763), 1:3200 (#sc-118, SCBT); and rabbit polyclonal anti-TrkB antibody (H-181), 1:250 (#sc-8316, SCBT). The NC membranes then were incubated for 1 h at RT with the secondary antibody (horseradish peroxidase (HRP) -conjugated anti-rabbit immunoglobulin G (IgG) antibody (Promega Corp., Madison, WI, USA) at 1:4000). All western blotting experiments were performed at least three times. All chemiluminescent signals were generated by incubation with the detection reagents (Immobilon; Merck KGaA) according to the manufacturer’s protocol. The relative intensities were determined using an LAS-3000 imaging system (FUJIFILM Wako Pure Chemical Corp.). Expression ratios were calculated against those of the Ponceau S staining intensities using Image J software (version 1.53a; National Institutes of Health, Bethesda, MD, USA).

### Statistical analysis

Data are expressed as means ± SE, and were analyzed using JMP 15.0 software (SAS Institute Japan Inc., Tokyo, Japan). P values of less than 0.05 were considered statistically significant. The detailed statistical methods are described in the individual figure captions.

## Results

### Far IR irradiation does not significantly attenuate body weight gain in AD mice

**Fig 2:**
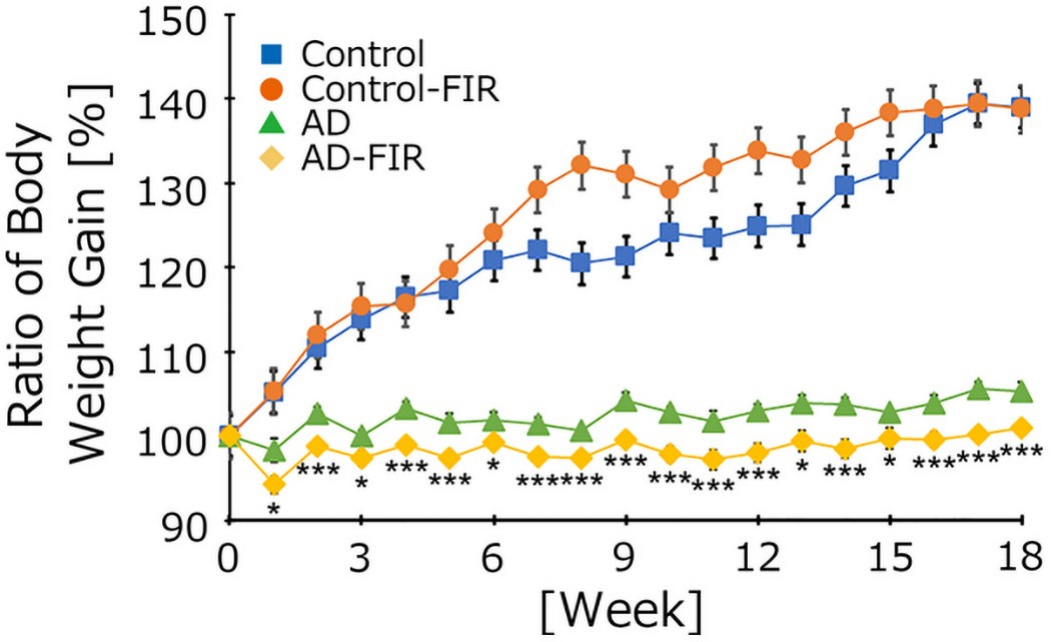
Relative body weights in treatment groups. Body weight was measured once per week for 5 consecutive months. Values were normalized to baseline (defined as 100%). Data are expressed as means ± SE (Control, *n* = 5; Control-FIR, *n* = 5; AD, *n* = 6; AD-FIR, *n* = 6). The timeline shows the treatment period. Statistical analysis was performed using a two-way analysis of variance (ANOVA). There was no significant difference in the presence or absence of IR light between the two mouse groups by two-way ANOVA. N.S., not significant (p ≥ 0.05). However, a direct comparison of the body weight ratios during each week of housing showed that the relative body weights of AD mice maintained in the presence of IR light were significantly lower than those of the AD group (*p < 0.05, **p < 0.01, ***p < 0.001; two-tailed non-paired Student’s *t*-test).

**Fig 3:**
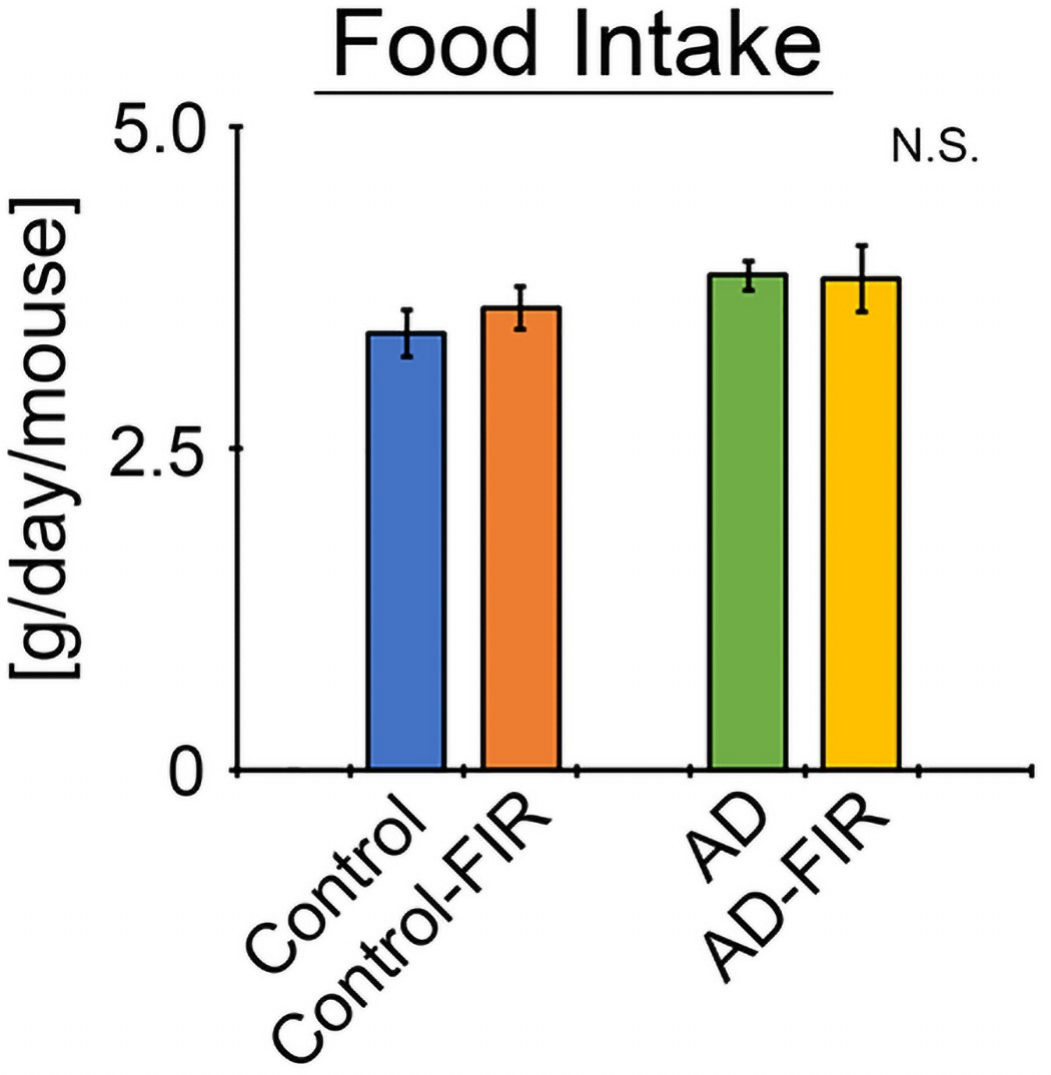
Daily mean food intake (g/day/mouse) in the presence and absence of FIR light. Food intake was measured once per week, and the mean per capita per diem consumption was calculated over the entire 5-month study period. Because all mouse groups were maintained on the same food pellets, daily caloric intake was not calculated separately. Data are expressed as means + SE (Control, *n* = 5; Control-FIR, *n* = 5; AD, *n* = 6; AD-FIR, *n* = 6). Comparisons in the presence or absence of FIR in both mouse groups were performed using a Tukey-Kramer’s test. N.S., not significant (p ≥ 0.05).

To clarify the biological effects of FIR light, we measured the body weights of control and AD mice that were housed with a fabric that either emitted FIR light or with one that did not. The relative body weight gain (normalized baseline) of AD mice was nominally (but not significantly) attenuated in animals housed with the fabric that generates FIR light (compared to those housed with control fabric) (Fig 2). However, there was no significant difference in the controls in the presence or absence of FIR light, and food intake weight did not differ in the presence or absence of FIR light between the two animal groups (Fig 3).

### Measurement of cognitive function of AD mice maintained in the presence or absence of FIR light

**Fig 4:**
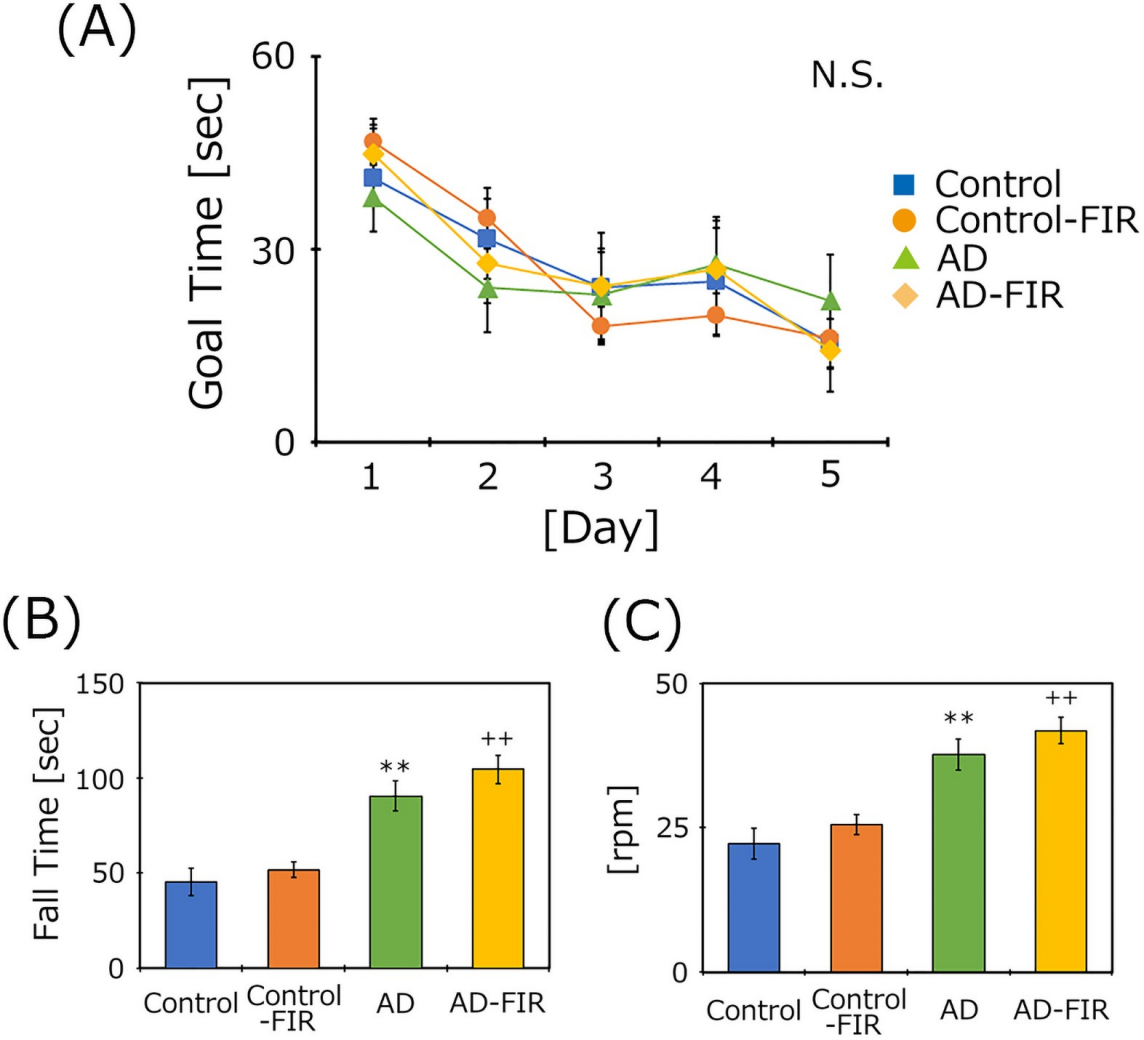
Assessment of cognitive and motor functions in the presence or absence of FIR light of the two mouse groups using (respectively) the Morris water maze test (A) and the Rota Rod test (B)(C). Data are expressed as means + SE (Control, *n* = 5; Control-FIR, *n* = 5; AD, *n* = 6; AD-FIR, *n* = 6). Data in Fig 4A were analyzed using two-way ANOVA; data in Fig 4B and 4C were analyzed using Tukey-Kramer’s tests. N.S., not significant (*p* ≥ 0.05). **p < 0.01 vs. Control, ^++^*p* < 0.01 vs. Control-FIR.

To clarify the effect of FIR light on the cognitive function of AD mice, we measured (at five months after the start of housing in the presence or absence of FIR irradiation) learning ability using the Morris water maze task (Fig 4A). The mice were subjected to swim tests four times per day on five consecutive days. The time to achieve the goal did not differ significantly in any mouse groups. The goal time on the final day of housing of the AD mice in the presence of the FIR irradiation was nominally lower than that in the unexposed AD group (housed without). At the same time points, motor function was assessed using the Rota Rod test (Fig 4B and 4C). The goal time and rod speed of AD mice were significantly higher than those of the controls in the presence or absence of FIR. There was no significant difference with or without FIR.

### Changes in serum parameters of AD mice in the presence or absence of FIR exposure

After the cognitive performance tests, the mice were bled and euthanized, and various segments of the brain were recovered. Serum was tested for multiple clinical chemistry parameters (Table 1). Some parameters of AD mice were significantly lower than those of the controls in the presence or absence of FIR. Notably, there was no significant difference in any of the parameters in the presence or absence of FIR exposure of the two mouse groups. The concentrations of bilirubin (both total and indirect) and, lipid parameters were nominally lower in the FIR-exposed AD group compared to the unexposed AD group, but these differences fell short of significance.

**Table 1:** Changes in serum parameters for control and AD mice maintained in the presence or absence of FIR light.

	Control	Control-FIR	AD	AD-FIR
Total protein [g/dL]	7.200±0.589	6.760±0.278	5.467±0.154	5.660±0.404
Albumin [g/dL]	4.240±0.194	4.280±0.102	3.600±0.106	3.580±0.229
Albumin/Globulin	1.425±0.111	1.550±0.161	1.933±0.092	1.740±0.068
Fe [μg/dL]	311.8±37.20	298.8±30.44	159.0±5.279#	161.8±11.67$
AST [IU/L]	292.75±57.523	222.67±44.86	83.00±18.50#	88.60±10.94
ALT [IU/L]	106.8±19.86	168.0±65.07	58.67±13.01	55.40±7.548
ALP [IU/L]	301.5±53.45	310.3±58.70	212.0±5.241	218.60±16.773
LDH [IU/L]	2006.0±171.92	1689.2±179.06	1291.2±69.491	1148.8±194.87
LAP [IU/L]	58.25±8.087	51.67±8.906	32.0±1.26	32.8±2.15
Total cholesterol [IU/L]	221.6±8.846	185.6±10.74	91.17±2.54##	80.40±8.611$$
Ester type cholesterol [IU/L]	176.2±6.606	144.8±5.886	79.33±1.838##	66.60±8.134$$
Triglyceride [mg/dL]	53.0±1.26	85.33±10.27	68.50±12.24	34.00±8.12$
E/T [%]	79.25±1.031	66.33±12.15	87.17±0.792#	82.20±1.59
HDL-cholesterol [mg/dL]	102.8±6.659	91.33±6.707	60.50±1.628#	52.80±6.651$
LDL-cholesterol [mg/dL]	31.00±4.123	27.33±4.551	7.00±0.45#	7.80±0.66$
Total bile acid [μmol/L]	9.00±2.39	9.60±1.72	2.66±0.33	4.00±1.15
Total bilirubin [mg/dL]	0.083±0.017	0.087±0.020	0.113±0.004	0.082±0.034
Indirect bilirubin [mg/dL]	0.078±0.012	0.080±0.016	0.083±0.012	0.058±0.019

^#^, ^##^ Control vs AD,

^$^, ^$$^Control-FIR vs AD-FIR.

Data are expressed as means ± SE (Control, *n* = 5; Control-FIR, *n* = 5; AD, *n* = 6; AD-FIR, *n* = 6). Data were analyzed by Tukey-Kramer’s test. AST, aspartate aminotransferase; ALT, alanine aminotransferase; ALP, alkaline phosphatase; LDH, lactic acid dehydrogenase; LAP, leucine aminopeptidase; E/T, ratio of cholesterol ester to total cholesterol; HDL, high density lipoprotein; LDL, low density lipoprotein.

### FIR exposure influences neurotrophic factor protein levels in the brains of AD mice

**Fig 5:**
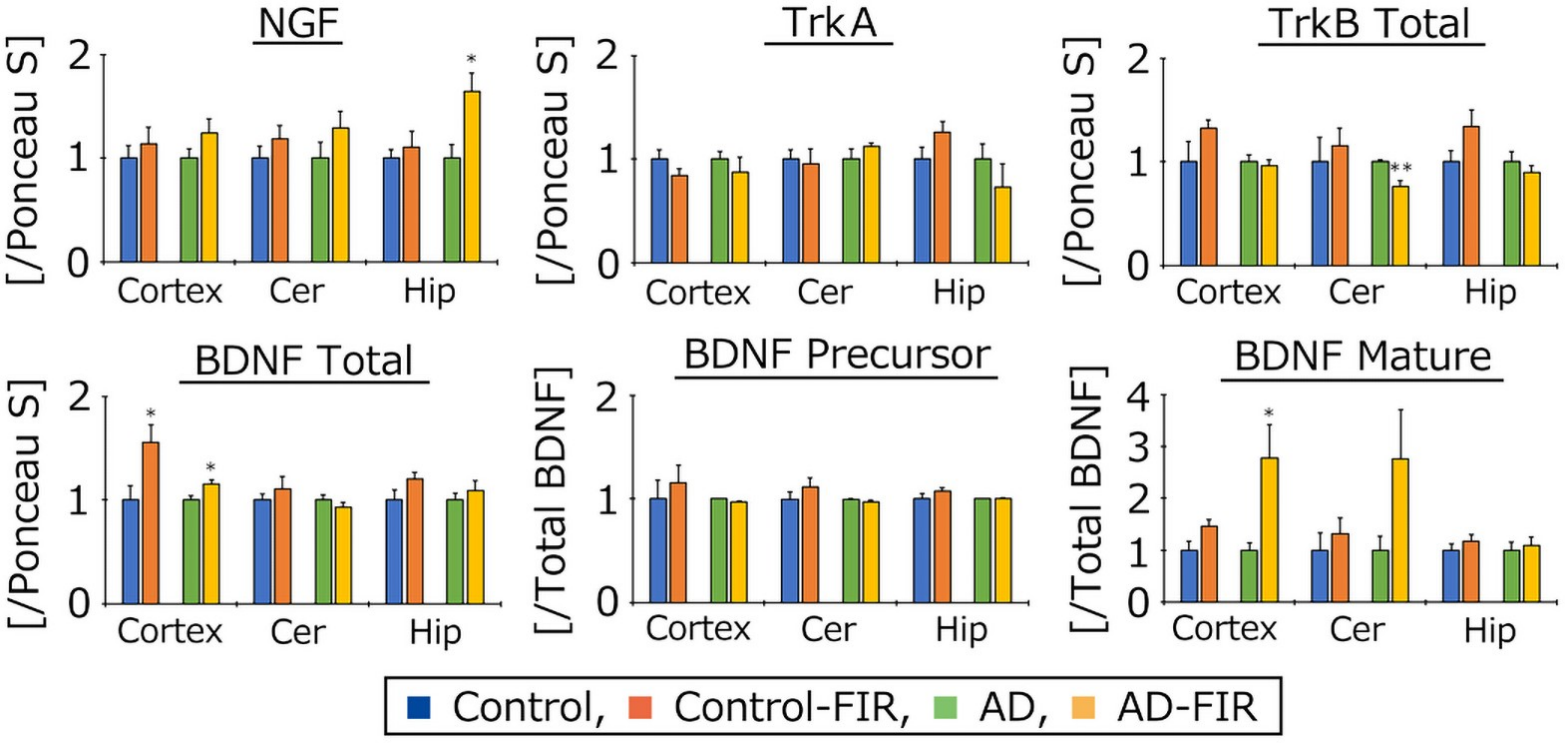
Western blotting analysis of the levels of each protein. Images of western blots for each protein examined in cortex (Cortex), cerebellum (Cer), and hippocampus (Hip) were scanned and digitized to determine band intensities, which were normalized to the intensity of staining by Ponceau S for the respective sample. Data are expressed as means ± SE (Control, *n* = 5; Control-FIR, *n* = 5; AD, *n* = 6; AD-FIR, *n* = 6). The data were analyzed using Tukey-Kramer’s tests. N.S., not significant (p ≥ 0.05). * *p* < 0.05, ** *p* < 0.01 vs. AD.

Finally, we checked the effects of FIR exposure on the levels of two major neurotrophic factors, NGF and BDNF. The levels of these factors, and of their respective receptors (TrkA and TrkB), were assessed in the cerebral cortex, cerebellum and hippocampus using western blotting (Fig 5). NGF and mature BDNF protein expressions were nominally higher in FIR-exposed mice compared to the unexposed mice. The NGF protein level in the hippocampus was significantly higher in the FIR-exposed AD mice than in the unexposed AD mice. However, the level of the NGF receptor, TrkA did not differ significantly in any mouse groups in any of the assessed brain regions. The level of mature BDNF and of total BDNF protein (where total = precursor + mature) protein expression in the cerebral cortex were significantly higher in the FIR-exposed AD mice than in the unexposed AD mice. The TrkB protein level in the cerebellum was significantly lower in the FIR-exposed AD mice than in the unexposed AD mice. However, the levels of the BDNF precursor protein did not differ significantly in any mouse groups for any of the tested brain regions.

## Discussions

### FIR exposure does not alter body weight gain in AD mice

The main purpose of this study was to clarify the potential health benefits of FIR light. Specifically, we assessed the effect of FIR light on AD-transgenic mice, given that FIR exposure may affect blood flow and change the oxygen concentration in small vessels in the brain. The blood flow volume gradually decreases during aging [16] and in AD [17]. The early stage of AD shows brain atrophy and neuronal cell death [18]. It is well known that amyloid plaques and hyperphosphorylation of tau protein induce neuronal cell death [4]. Decreased blood flow leads to decreased oxygen levels and under-nutrition, accelerating the progression of AD.

We monitored body weight and food intake in mice during aging from 8 to 12 months, over the course of 5 months of IR exposure (or lack thereof). The two groups differed based on the presence of a swatch of experimental (IR-emitting) or control (non-emitting) fabric in their cages, with the two types of fabric differing based on the former being impregnated with an ore/compound that emits FIR light. The relative body weight gain (normalized to baseline) in FIR-exposed AD mice was nominally (though not significantly) attenuated compared to that of the unexposed AD mice. In contrast, food intake weight did not differ significantly any of the mouse groups. Thus, the FIR-exposed AD mice consumed a similar amount of food as the unexposed AD mice, but gained (nominally) less weight. This difference may be attributable to increased metabolic function. It has been reported that exposure to FIR light by leg hyperthermia, saunas, or heat therapy in humans relieves the symptoms of diabetes [19]. In other work, Masuda et al. showed that FIR light sauna therapy decreases 8-epi-prostaglandin F(2α) (PGF_2α_) levels in humans [20]. Under standard conditions (i.e., in the absence of IR exposure), the ratio of body weight gain of AD mice tends to be lower than that of age-matched controls, and lipid data also tend to worsen in AD mice compared to the controls. Using a mouse model, Washington et al. reported that ceramic particles absorb heat emitted from the body, and then re-emit the associated thermal energy back into the body as IR radiation [21]. In separate work, we showed (as confirmed by an external verification organization) that the fabric used in the present study emits FIR light with wavelengths of 8 to 10 μm. Warming of the cage floor to approximately 37°C would also be expected to cause the fabric to absorb heat, which may in turn be released as FIR light, to which the animals would be exposed. However, mice prefer cooler conditions of approximately 23°C. It is difficult to explain this apparent conflict in housing conditions. We have not defined the detailed mechanism of the nominal weight gain attenuation effect on the AD mice of the FIR light that was shown in this study. It may be much better to use high fat-diet-treated mice for assessing the weight gain attenuation effects of FIR exposure. Currently, we are monitoring the respiratory quotients in FIR-exposed mice to assess metabolism. Clearly, further investigation will be needed to clarify this effect and its mechanism.

### FIR exposure enhances NGF levels in the hippocampus of AD mice

To clarify the effects of FIR exposure on cognitive function, we assessed learning ability using the Morris water maze task. This test measures the time taken by mice to reach a hidden acrylic platform [13]. The test times did not differ significantly for AD mice housed in the presence or absence of FIR light, although the times on the final day of testing were nominally (though still not significantly) lower in the FIR-exposed AD mice compared to the unexposed AD mice. Learning ability in the Morris water maze is known to decrease significantly as mice age, and to be lower in AD animals compared to age-matched controls [14]. We confirmed that the swimming speed did not differ between the two groups (Control, 0.1259 m/sec; Control-FIR, 0.130 m/sec, AD, 0.1282 m/sec; AD-FIR, 0.1378 m/sec), nor did motor function as assessed using the Rota Rod apparatus. These results showed that FIR exposure did not alter cognitive or motor function in AD mice. Purushothuman et al. [22] reported that exposure to near infrared light attenuates amyloid beta deposition and neurofibrillary tangles in the cerebellum region of APP/PS1 and K3 transgenic mouse models. Moreover, some researchers have pointed out the beneficial biological effects of microwaves against neurodegenerative disorders [23,24]. In this study, we used the 5xFAD strain. This strain contains five mutations, and symptoms tend to appear quickly and strongly compared to other AD transgenic models. Official information from the Jaxon Laboratory (https://www.jax.org/strain/006554) indicates that 1.5 months of age is when beta amyloid accumulation is first observed. In our experiments, the starting age of AD-transgenic mice was 8 months old. Previously, we checked beta amyloid deposition in the hippocampus of a 6-month-old mouse of the same strain (S1 Fig). It is possible that beta amyloid aggregation has already occurred considerably by 8 months of age, and the experiment started late. However, the literature indicates that it is exceedingly rare to demonstrate a correlation between brain/cognitive function and a specific treatment. Science-based evidence for successful treatments remains difficult to obtain in this research field, an observation that is an ongoing challenge.

To assess whether FIR exposure affects brain function, we measured the brain levels of two specific neurotrophic factors and their cognate receptors in AD mice. Notably, we found that the levels of NGF protein in the hippocampus, and of mature BDNF in the cerebral cortex, were significantly higher in the FIR-exposed AD mice than in the unexposed AD mice. Both neurotrophic factors play important roles in the maintenance of neuronal activity, and depletion of these factors is intimately related to progressive cognitive decline in AD [25,26]. NGF/TrkA signaling also is attenuated in rodent models of aging, including cognitive decline via impairment of cholinergic function [27]. Administration of exogeneous BDNF has been shown to improve cognitive function in AD mice [28]. The depletion of neurotrophic factors may induce impairment of many neuronal functions, such as synaptic [29], dendritic [30], and axonal degeneration [31]. In the present study, FIR light did not influence cognitive function in AD mice. However, after exposure to FIR light, the concentration of selected neurotrophic factor levels clearly changed in some brain regions in AD mice. Exposure of AD mice to FIR light for longer intervals may result in better differentiation from unexposed animals. Further investigation will be needed to elucidate the possible beneficial effects of FIR exposure in AD mice, including the incorporation of changes in the experimental protocol and exposure period.

### FIR exposure does not change serum parameters in AD mice

Assessment of terminal serum chemistry in the AD mice revealed that most parameters did not differ significantly between the FIR-exposed mice and the unexposed mice. Some parameters in the AD mice were significantly lower than in the control mice in the presence or absence of FIR light. However, the levels of TGs and the E/T ratio in the FIR-exposed AD mice were nominally (but not significantly) lower than those in the unexposed AD mice. It is possible that the decrease of TG levels may depend on the attenuation of weight gain in the FIR-exposed AD mice. The E/T ratio is also strongly correlated to the cholesterol metabolism. HDL cholesterol was nominally decreased in the FIR-exposed AD mice compared to the unexposed AD mice, though this effect did not achieve significance. Together, these results indicated that FIR exposure may influence liver function and lipid metabolism. However, the levels of AST and ALT did not differ in FIR-exposed mice compared to the unexposed AD mice. Further investigation is needed to clarify the mechanism. A review of the literature did not reveal previous references showing a relationship between FIR exposure and changes in liver function including TG levels and E/T ratio. If FIR exposure does affect blood flow, these parameters might be improved in AD mice.

## Conclusions

In this study, we examined the possible biological effects on AD mice of long-term exposure to FIR light. Notably, neither cognition nor motor function in AD mice was improved after FIR exposure in our experimental model. The destiny of this AD mouse model already has been decided by genetic engineering. It may be very difficult to alter this fate by non-invasive methods. In that respect, it may be much better to use normal-aged mice for testing the possible biological effects of FIR exposure. We also used normal mice in this study, but the number of animals was small and could not clearly detect the biological effect of FIR exposure. However, changes were seen in body weight and in the levels of neurotrophic factors in specific brain segments in AD mice exposed to FIR light. Although there is room for improvement in our experimental method including the number of animals, exposure period, and housing conditions, some biological parameters for AD mice clearly were altered in our experiment. FIR light is already used clinically. We hope our basic data will be of use in the development of future research and potential treatments.

## Supporting information

S1 Fig Aβ(1–40) expression in the hippocampal region of a 6-month-old AD-transgenic mouse. After circulation fixation, we obtained sections and stained them using anti-Aβ(1–40) antibody. The scale bar is 20 μm.
(TIF)
