# Supplementary figures and images for: Effects of far infrared light on Alzheimer’s disease-transgenic mice
Source: PLoS One. 2021 Jun 17;16(6):e0253320. doi: 10.1371/journal.pone.0253320 (PMC8211253; doi:10.1371/journal.pone.0253320)

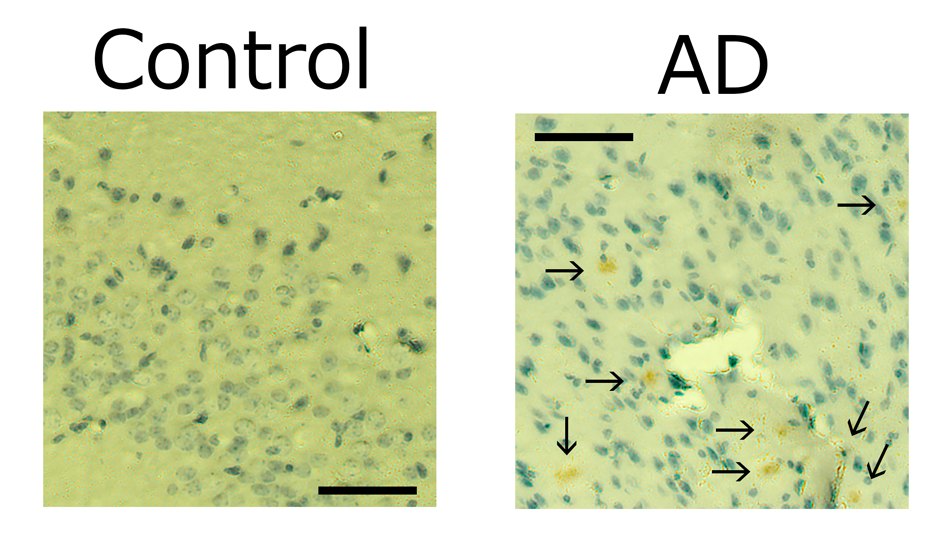

Supplement: S1 Fig — After circulation fixation, we obtained sections and stained them using anti-Aβ(1–40) antibody. The scale bar is 20 μm. (TIF) [file pone.0253320.s001.tif]
